# Re-Arranging the Puzzle between the Amyloid-Beta and Tau Pathology: An APP-Centric Approach

**DOI:** 10.3390/ijms25010259

**Published:** 2023-12-23

**Authors:** Florence Haut, Elentina K. Argyrousi, Ottavio Arancio

**Affiliations:** 1 Taub Institute for Research on Alzheimer’s Disease and the Aging Brain, 630 West 168th Street, P&S, New York, NY 10032, USA; fh2283@cumc.columbia.edu (F.H.); ea2693@cumc.columbia.edu (E.K.A.); 2 Department of Medicine, Columbia University, New York, NY 10032, USA; 3 Department of Pathology and Cell Biology, Columbia University, New York, NY 10032, USA

**Keywords:** amyloid-beta, Tau, amyloid-precursor protein, amyloid cascade hypothesis

## Abstract

After several years of research in the field of Alzheimer’s disease (AD), it is still unclear how amyloid-beta (Aβ) and Tau, two key hallmarks of the disease, mediate the neuropathogenic events that lead to AD. Current data challenge the “Amyloid Cascade Hypothesis” that has prevailed in the field of AD, stating that Aβ precedes and triggers Tau pathology that will eventually become the toxic entity in the progression of the disease. This perspective also led the field of therapeutic approaches towards the development of strategies that target Aβ or Tau. In the present review, we discuss recent literature regarding the neurotoxic role of both Aβ and Tau in AD, as well as their physiological function in the healthy brain. Consequently, we present studies suggesting that Aβ and Tau act independently of each other in mediating neurotoxicity in AD, thereafter, re-evaluating the “Amyloid Cascade Hypothesis” that places Tau pathology downstream of Aβ. More recent studies have confirmed that both Aβ and Tau could propagate the disease and induce synaptic and memory impairments via the amyloid precursor protein (APP). This finding is not only interesting from a mechanistic point of view since it provides better insights into the AD pathogenesis but also from a therapeutic point of view since it renders APP a common downstream effector for both Aβ and Tau. Subsequently, therapeutic strategies that act on APP might provide a more viable and physiologically relevant approach for targeting AD.

## 1. Introduction

Alzheimer’s disease (AD) was first described by German neuro-psychiatrist Alois Alzheimer in 1906, and till today it represents the most common form of dementia in the elderly—a progressive, neurodegenerative disease characterized by synaptic alterations, neuronal loss, and cognitive dysfunction [1]. If present trends continue and the aging population continues to grow, the burden of AD will eventually overwhelm the healthcare system. Currently, researchers project that the worldwide prevalence of AD will increase to roughly 110 million cases by 2050 [2]. At the pathophysiological level, AD is characterized by the presence of extracellular plaque formations containing amyloid-beta (Aβ) and intraneuronal fibrillary tangles containing hyperphosphorylated Tau [3]. Despite the first discovery of these histological hallmarks, the exact cause of the pathological cascades that triggers the production of Aβ and hyperphosphorylated Tau is yet unclear. Genetic mutations are generally linked with early-onset familial AD (FAD) in which the individuals exhibit signs of the disease before the age of 65. These genetic mutations are found within the genes of amyloid precursor protein (*APP*), presenilin 1 (*PSEN1*), and presenilin 2 (*PSEN2*). Late-onset AD affects individuals after the age of 65 and is associated with the presence of the ε4 allele in the apolipoprotein E (*APOE*) gene [4,5]. Besides genetic risk factors, acquired risk factors, such as cerebrovascular diseases, type 2 diabetes, hypertension, obesity, dyslipidemia, stress, depression, sleep deprivation, and marital status, have also been associated with the development of AD [6,7]. Despite over a century of research aiming to understand the etiology of AD and subsequently suggest possible treatments, it has become more apparent that our current knowledge of the disease is insufficient for providing an efficient therapeutic approach. In this respect, the current AD-approved treatments could either alleviate early symptoms of the disease or partially slow down its progression with no knowledge of long-term efficacy and the risk of severe side effects [8,9]. Accordingly, the main hypothesis aiming to describe the pathological events occurring in AD referred to as the “Amyloid Cascade Hypothesis”, has been challenged by recent findings. The “Amyloid Cascade Hypothesis”, that has prevailed in the AD field for almost 30 years, suggests that Aβ deposits are the initiators and drivers of AD. Specifically, Aβ pathology is placed above Tau pathology, both chronologically and mechanistically, suggesting that the early presence of Aβ acts as a catalyst for initiating Tau pathology. From a therapeutic point of view, this mechanistic model in which Aβ triggers Tau pathology led scientists to speculate that targeting Aβ at early disease stages might be the most promising treatment of AD. Indeed, several healthcare companies have developed drugs that target Aβ, but the results have not been promising. The failure of Aβ-targeting treatments was attributed to the late onset of therapy initiation at a stage in which Aβ triggered Tau pathology, thus rendering it the main toxic factor in the progression of the disease. Subsequently, this explanation led the interest of the scientific community towards unraveling the pathological function of Tau in AD, focusing on Tau-related therapeutic interventions. Nevertheless, recent studies propose that Aβ and Tau act independently and not in sequence, as was previously suggested, for inducing neurotoxicity in AD. Importantly, several studies have shown that Aβ and Tau affect neuronal function by acting on the same targets (for a review see [10]).

Unraveling the relationship between Aβ and Tau, as well as their downstream effectors for spreading the disease, is of paramount interest for the development of efficient therapeutic interventions. In this respect, drugs that act on both Aβ and Tau or a common downstream target might be the most promising approach in treating the disease, since drugs that act either on Aβ or Tau cannot provide a holistic effect.

In the present review, we summarize current studies related to the role of APP in the pathogenesis of AD, as well as its physiological role in normal neuronal function. Additionally, we discuss the role of Aβ and Tau in AD pathogenesis as well as recent Aβ- and Tau-targeting therapeutic approaches with an emphasis on pitfalls of treatments aimed at targeting Aβ and tau pathology. Aiming to better understand the pathological events associated with the disease, we highlight studies showing that Aβ and Tau do not require each other to trigger AD. In this respect, we discuss recent findings showing that APP is a common downstream mediator for Aβ and Tau, mediating their neurotoxic functions. Importantly, we suggest a new model in which Aβ and Tau act in parallel and through APP to induce plasticity and memory decline in AD. Subsequently, we propose that unraveling the complex interplay between APP, Aβ, and Tau might be the key to the development of novel treatments that target AD.

## 2. Amyloid Precursor Protein: Enzymatic Cleavage and AD Pathogenesis

The amyloid plaques that constitute one of the main pathological AD hallmarks are composed of aggregated Aβ peptide that is generated during cleavage of APP. Considering the important role of APP in AD pathology, there are several studies regarding its complicated enzymatic cleavage, its impact on the progression of AD, as well as its interaction with Aβ and Tau.

APP is a single-pass transmembrane protein widely expressed in the central nervous system and some peripheral tissues [11]. It has a large extracellular amino-terminal membrane domain, a transmembrane domain, and a short carboxy-terminal cytosolic tail. The gene for APP is located on the long arm of chromosome 21 at position 21q11.2 [11,12]. Differential cleavage of APP could result in amyloidogenic or non-amyloidogenic processing. Non-amyloidogenic processing occurs when APP undergoes sequential cleavage by α- and γ-secretase, releasing the following two fragments: a soluble APP fragment alpha (sAPPα), which is released from the cell membrane; and a C-terminal fragment (CTF), called CTFα, which is a carboxy-terminal fragment that remains anchored to the plasma membrane. Subsequently, CTFα is cleaved by γ-secretase, a multiprotein complex composed of four proteins, including presenilin 1, presenilin 2, alpha-1, and nicastrin, to give rise to the extracellular small p3 peptide and the intracellular peptide AICD/AID (amyloid intracellular domain). Conversely, in the amyloidogenic pathway, the initial cleavage of APP by β-secretase-1 (BACE-1) gives rise to the extracellular sAPPβ fragment and the transmembrane CTFβ fragment. Cleavage of CTFβ by γ-secretase results in the production of Aβ and AICD/AID [13]. More recently it was identified that APP could be cleaved in vitro by other secretases, adding to the complexity of APP processing. Cleavage of APP by asparagine endopeptidase (AEP) or δ-secretase and η-secretase have been also linked to Aβ production. In more detail, AEP or δ-secretase cleaves APP in 2 sites in the ectodomain, resulting in 3 soluble fragments of APP and a CTFδ fragment. CTFδ is subsequently cleaved by BACE-1 and γ-secretase, producing AICD/AID and Aβ [14]. Although the exact role of δ-secretase in AD is not fully known, there is evidence showing that knock-out (KO) of the AEP gene could ameliorate pathology and memory deficits in 2 animal models of AD [14]. Similar to δ-secretase, cleavage of APP by η-secretase has been also associated with AD pathogenesis. Cleavage of APP by η-secretase results in the production of the sAPPη and the CTFη fragments. The latter could be cleaved by α- or β-secretase leading to the release of the Aη-α or Aη-β fragments, respectively [15,16]. The remaining membrane-bound CTF fragment could be cleaved by γ-secretase for the production of AICD/AID and p3 or Aβ fragments. Interestingly, Aη-α was shown to impair synaptic plasticity in hippocampal slices ex vivo and to reduce neuronal activity in vivo, while Aη-β fragments have no effects in these measurements [16]. The involvement of η-secretase cleavage of APP in AD pathogenesis was supported by the finding that η-secretase and CTF-η are found within dystrophic neurites in close proximity to amyloid plaques [16,17].

APP processing takes place in different locations with APP cleavage by α-secretase occurring at the plasma membrane and APP cleavage by BACE-1 occurring in different intracellular components [18,19]. The majority of APP is located in the Golgi complex, where it will either be inserted at the plasma membrane or directed to lysosomes for degradation [20]. Several studies have shown that α-secretases mainly reside at the plasma membrane, where APP is cleaved in the non-amyloidogenic pathway, while BACE-1 is located in the Golgi complex and endosomes, where APP is cleaved in the amyloidogenic pathway [21,22]. In turn, the generation of Aβ is completed by cleavage of CTFβ by γ-secretase which is also mainly located in the Golgi complex and endosomes [11]. Thereafter, the majority of Aβ is produced in the Golgi complex and endosomes. The exact function of APP is still not fully understood, but several studies suggest that APP participates in important neuronal functions including neuronal survival, neurite outgrowth, enhanced synaptogenesis, and synaptic plasticity [23]. It is also suggested that APP could act as a receptor, transducing signals extracellularly, but this hypothesis is not supported by strong evidence. An important aspect of APP function is its participation in synaptic function. Specifically, APP is transported from the axons to synaptic terminals via anterograde axonal transport in tubular vesicles [24,25]. Cleavage of APP in synaptic terminals, results in the deposition of Aβ and other components of APP processing in the synapses [26]. Upon generation, Aβ monomers are either secreted into the extracellular space or retained intracellularly where they aggregate (for a review see [27]). Interestingly, intraneuronal accumulation of Aβ precedes the formation of amyloid plaques and is correlated with neuronal loss in an AD mouse model [28], as well as altered neuronal morphology and neuroinflammation [29]. Aggregation of Aβ is facilitated by the β-sheet conformation that promotes hydrophobic interactions [30,31]. Although insoluble Aβ fibrils are the histopathological indicators of AD, several studies suggest that soluble Aβ oligomers (oAβ) induce neurotoxicity [32,33,34]. This is supported by the weak association between amyloid plaques and memory impairments in mice expressing human APP (hAPP) and conversely by the observation of memory impairments in AD mouse models that lack amyloid depositions [34,35,36]. Importantly, it was shown that artificially prepared or naturally secreted oAβ could impair synaptic plasticity, assessed in long-term potentiation (LTP) recordings both in vitro and in vivo [37,38].

Although full-length APP is closely associated with the production of Aβ, it also plays an essential role during development and adult life (for a review see [39]), facilitating differentiation and growth of neuronal cells [25]. The function of APP in the brain is mainly associated with presynaptic functions related to the regulation of presynaptic vesicles and the subsequent release of neurotransmitters. In more detail, analysis of synapses in double KO (DKO) mice for APP and the related APP-like protein 2 (APLP2) showed a reduction in the synaptic vesicle density, active zone size, and number of docked vesicles [40]. Additionally, a lack of APP leads to a reduction of synaptic vesicle proteins, such as synaptophysin, synaptotagmin-1, and SV2A at the presynaptic release sites, having an impact on neurotransmission [41]. A later study from Fanutza et al. showed that the interaction of APP with the neurotransmitter release machinery facilitates neurotransmitter release from the presynaptic cell [42]. The same study showed that AAP interacts with synaptic vesicles via the cytosolic (JCasp) and the intravesicular (ISVAID) domains leading to an increase or decrease in neurotransmitter release, respectively [42]. Cleavage of APP by α-secretase or BACE-1, occurring within the ISVAID domain, diminishes the interaction of ISVAID with synaptic vesicles, and results in increased release probability of neurotransmitters [43]. This phenomenon is specific for glutamatergic synaptic vesicles, suggesting that cleavage of APP could modulate excitatory synaptic transmission [43]. A study from Lee et al. shed more light on the function of APP in the adult brain by creating an APP/APLP1/APLP2 conditional triple KO (cTKO) mice in which APP is deactivated in excitatory neurons in the forebrain [44]. This study showed that although these mice do not exhibit neurodegeneration, they have profound impairments in synaptic plasticity and memory. 

The changes in synaptic plasticity indicate that the loss of function of APP could lead to hyperexcitability, indicating that the presence of APP in the adult brain is important for preventing toxic hyperexcitability [44]. These results are in agreement with the previously suggested role of APP in neuronal excitability [45,46]. Moreover, they suggest the possibility that scenarios alternative to the amyloid cascade hypothesis might be involved in AD pathogenesis with full-length APP or different fragments derived from its cleavage causing the disease symptoms.

APP could also participate in synaptic plasticity through the function of AICD/AID, the intracellular peptide that is generated after γ-secretase cleavage. In more detail, it was shown that AICD/AID forms complexes with the multidomain adaptor protein Fe65 and the histone acetyltransferase Tip60, which is subsequently transferred to the nucleus where it acts as a transcription factor [47,48]. Importantly, it was shown in vitro that AICD/AID participates in phosphoinositide-mediated intracellular calcium (Ca^2+^) signaling [49]. This latter observation raises the possibility that AICD/AID participates in the expression of genes related to the modulation of intracellular Ca^2+^ levels by altering the sensitivity of the phosphoinositide Ca^2+^ signaling pathway [49]. Since these first discoveries, several other studies have aimed at unraveling the AICD/AID target genes that are relevant to AD (for a review see [50]). An interesting discovery is that AICD/AID stimulates the activity of the glycogen synthase kinase 3β (GSK3β) in vivo without altering its transcription or translation [51]. The latter finding was attributed to post-translational modifications and provides a link between APP and the Tau pathology since hyperphosphorylation of Tau in AD is attributed to enhanced GSK3β activity [52]. Interestingly, in a study by Aplin et al., it was shown that GSK3β could phosphorylate AICD/AID at the T688 residue that is implicated in the development of AD [53].

Several reports have explored the role of sAPPα in neuroprotection, highlighting its therapeutic potential for AD (for a review see [54]). In addition, sAPPα could be used as a diagnostic marker for the disease, since sAPPα levels are significantly reduced in the cerebrospinal fluid (CSF) and platelets of AD patients [55,56,57,58]. Interestingly, reduced levels of sAPPα in the CSF were correlated with memory impairments in AD patients and aged mice [59,60], suggesting the important role of sAPPα in cognitive function [61]. Regarding the neuroprotective action of sAPPα, it was shown in primary hippocampal cell cultures that sAPPα could protect against Aβ-induced toxicity via compensating elevated intracellular Ca^2+^ levels and reactive oxygen species [62]. Along these lines, overexpression of sAPPα could rescue plasticity and memory impairments observed in APP-deficient mice [63] or mice exhibiting AD pathology [64]. In more detail, sAPPα overexpression reduced Aβ levels and enhanced clearance of amyloid plaques through microglia recruitment [64]. Another study showed that sAPPα could attenuate Aβ production via modulating APP cleavage. The latter function was mediated via direct binding of sAPPα to BACE-1 [65]. Interestingly, it was shown that overexpression of sAPPα in AD mice leads to a reduction of GSK3β activity and Tau phosphorylation, underscoring the relationship between APP cleavage and Tau pathology [66]. It was also shown that overexpression of sAPPα in the hippocampus of conditional KO (cDKO) that lack APP and the APLP2 could ameliorate synaptic plasticity impairments, dendritic branching abnormalities and spatial memory deficits [67]. The effect of sAPPα seemed to involve the activation of the α7 nicotinic acetylcholine receptor (α7-nAChR). In this respect, it was shown in oocytes overexpressing α7-nAChRs that sAPPα could act as a positive allosteric modulator for the receptor, suggesting that the effect of sAPPα on synaptic plasticity is mediated via the cholinergic signaling [67]. In addition, sAPPα could participate in synaptic plasticity via modulation of the glutamate signaling, since sAPPα could rescue age-related plasticity impairments via activation of the N-methyl-D-aspartate receptor (NMDAR) [68].

Although APP can be phosphorylated at several sites, phosphorylation at T688 in the intracellular domain was extensively studied due to its significance in several aspects of APP function. In this respect, it is controversial whether phosphorylation at T688 enhances or diminishes interaction between AICD/AID and Fe56. Despite this discrepancy, several studies conclude that phosphorylation at T688 could exacerbate AD pathology. A study from Ando et al. has shown that phosphorylation at T688 reduces interaction with F65 by inducing conformational changes in the cytoplasmic domain that contains the F65-binding motif. The same study also showed that overexpression of F65 could reduce the secretion of Aβ in HEK293 cells [69]. Thereafter, it was proposed that phosphorylation of APP at T688 could alter its interaction with F65, eventually affecting the metabolism of APP in neurons. A later study from Chang et al. showed that phosphorylation at T688 facilitates interaction between AICD/AID and F65, promoting its translocation to the nucleus and inducing cytotoxicity, possibly via stimulating transcription of GSK3β. This later study also highlighted the important role of T688 phosphorylation in AD pathology by demonstrating that AD human brains and AD animal models exhibit increased levels of T688, GSK3β, and Tau phosphorylation [70]. Subsequently, a reduction in T688 phosphorylation by specific inhibitors or genetic mutations at this site could ameliorate neurotoxicity in vitro [70]. Along the same lines, it was suggested that T688 phosphorylation promotes preferential cleavage of APP by BACE-1. Interestingly, the blockage of T688 phosphorylation by mutation of T to A or by kinase inhibitors was able to reduce Aβ production in vitro [71]. The therapeutic potential of targeting T688 phosphorylation in AD was further underscored by a study from Lombino et al., showing that genetic mutation from T to A protected against synaptic plasticity and memory impairments in an FDD_KI_ mouse model of dementia [72]. It is important to notice that this mutation did not affect the biological function of APP during development [73], indicating that targeting T668 phosphorylation might be a safe therapeutic option for the treatment of AD [72].

## 3. Aβ: AD Pathogenesis, and Drawbacks of Anti-Aβ Therapies

Since AD was first described, there has been a plethora of studies examining the detrimental role of Aβ in a series of pathological events leading progressively to cognitive impairment. As mentioned before, APP is cleaved by BACE-1 and γ-secretase in endocytic compartments, the Golgi apparatus, and the endoplasmic reticulum. The length of the Aβ peptide depends on the site of y-secretase cleavage on CTFβ, varying from 38–43 amino acids. The most predominant form of the Aβ peptide is Aβ40 [74]. Although Aβ42 is found in lower concentrations, it has received more attention regarding its role in AD due to its tendency to form aggregates [74]. Additionally, several studies suggest that an increased Aβ42/Aβ40 ratio correlates with early-onset FAD (for a review see [75]). These observations are not surprising since FAD is associated with mutations in *APP*, *PSEN1*, and *PSEN2* genes [76]. Several mutations in the *APP* gene have been identified within the proteolytic cleavage domain and have been shown to increase the production of Aβ42. Specifically, it was shown in vitro that mutations in the APP sequence within the γ-secretase cleavage domains could increase the Aβ42/Aβ40 ratio [77]. The important role of Aβ42 in AD pathogenesis and subsequent diagnosis has been emphasized by studies showing that the Aβ42/Aβ40 ratio in the plasma or CSF could be used as a biomarker for diagnosing AD and distinguishing AD from other tauopathies [78,79].

Although a great number of studies have been dedicated to unraveling the pathological function of Aβ, more recent studies have shown that Aβ plays an important physiological role in the healthy brain, facilitating neuronal transmission [80]. The biphasic role of Aβ in synaptic transmission depends on its concentration and is an integral part of neuronal activity. In more detail, it was shown that neuronal activity facilitates cleavage of APP by β-secretase, subsequently leading to elevated Aβ production. The increased presence of Aβ suppresses neuronal activity and reduces Aβ production, creating a neuroprotective feedback mechanism [81]. Another study has shown that increased activity leads to increased APP endocytosis into endosomes, where it is cleaved via the amyloidogenic pathway [82]. Importantly, increased activity promotes the secretion of Aβ into the extracellular space via enhanced synaptic vesicle exocytosis [83].

In alignment with the physiological function of Aβ, it was shown that the peptide can act as an antimicrobial peptide in the brain. Specifically, it was shown in vitro that synthetic Aβ could protect against influenza A and the herpes simplex virus [84,85]. These findings were verified in vivo, showing that Aβ oligomerization is part of the innate immune response to microbial infections [86]. Nevertheless, prolonged exposure to a pathogen could lead to excessive accumulation and seeding of Aβ, shifting its function from neuroprotective to pathological (for a review see [87]).

Endogenous Aβ is needed for intact synaptic plasticity and memory formation [88,89], and blockage of its production in the healthy brain results in impairments of these mechanisms [89]. This notion is strengthened by studies showing that mimicking the physiological concentrations of Aβ in the brain by administration of picomolar concentrations of the peptide could enhance LTP and memory [90]. Better insight into the physiological functions of picomolar concentrations of oligomeric Aβ42 (oAβ42) was given by a later study from Gulisano et al., showing that oAβ42 could modulate several presynaptic and postsynaptic mechanisms [91]. In more detail, the extracellular application of picomolar concentrations of oAβ42 promoted the release of neurotransmitters from the presynaptic cell. In addition, at the postsynaptic cell, oAβ42 increased postsynaptic density and enhanced expression of plasticity-related proteins, promoting the formation of long-term potentiation (LTP) and long-term memories. The effects of oAβ42 were maintained even in the absence of endogenous APP [91]. Importantly, the action of oAβ42 at picomolar concentrations was dependent on the presence of α7nAChRs, since blockage of α7nAChRs abolished the effect of oAβ42 on presynaptic and postsynaptic mechanisms [92].

Besides the important physiological function of picomolar concentrations of Aβ, it has been widely established that high concentrations of the peptide can lead to the derangement of synaptic mechanisms, leading eventually to neuronal loss and memory impairments. Accumulation of Aβ in the extracellular space could cause loss of spine and synapses, altering synaptic transmission and plasticity [93]. Although insoluble fibrillar species of AD are present in senile plaques, breakthrough studies in the field of AD have established that soluble species ranging from dimers to high-molecular-weight oligomers would affect synaptic plasticity, as depicted with impairments in LTP [94]. The notion that oAβ is the toxic species in AD was supported by findings showing the presence of oAβ in the CSF of humans even decades before the onset of AD [95]. The establishment of oAβ, but not monomers or fibrils, in the center of AD pathology, changed our understanding of the disease and subsequently, the therapeutic interventions that were originally focused on reducing plaque burden. Through the years, there have been several amyloid-centric therapeutic approaches aiming at restoring the balance between Aβ production and Aβ clearance from the brain [96]. These therapeutic strategies fall into three main categories. First, small molecules that inhibit Aβ production by inhibiting β- or γ-secretases were under thorough investigation. An oral BACE-1 inhibitor, verubecestat, that showed promising results in lowering Aβ levels in the CSF of rodents, non-human primates, and AD patients, was proven unsuccessful since it not only failed to improve cognition in patients with mild-to-moderate AD but also caused a series of adverse side effects [97]. Similarly, despite the promising results in reducing Aβ production, clinical trials with the γ-secretase inhibitors semagacestat and avagacestat were discontinued due to serious side effects, such as the development of skin cancer and the worsening of cognitive performance [98,99]. Another strategy in combating Aβ pathology involved active or passive immunization with antibodies acting against the Aβ peptide. In animal models, administration of antibodies against Aβ proved to be a promising strategy in preventing the formation of senile plaques [100]; this was probably due to the activation of microglial cells that clear plaques via phagocytosis, thus promoting peptide degradation [101]. Subsequently, Aβ is redistributed from the brain to the plasma as depicted by increased levels of Aβ in the plasma after active immunization [102]. Nevertheless, clinical trials with active (AN-1792, CAD-106, and vanutide cridificar) or passive immunization (bapineuzumab, solanezumab, crenezumab, and gantenerumab) were unsuccessful and side effects were reported. For instance, treatment with gantenerumab, a fully human anti-Aβ monoclonal antibody, reduced levels of amyloid plaques after 116 weeks of treatment, but did not improve cognitive performance in patients with early AD [103]. Nevertheless, two other anti-Aβ strategies showed some beneficial effects in improving cognitive performance. In more detail, a human monoclonal antibody, aducanumab, gained interest since intravenous infusions of aducanumab over the course of a year reduced levels of Aβ in the brain and slowed cognitive decline [104]. Nevertheless, concerns have been raised regarding its safety and tolerability, since during the clinical trials, 41% of the patients exhibited brain swelling and bleeding that was mitigated after lowering the dose. Despite this observation deeming further development of aducanumab for the treatment of AD, the drug was approved in 2023 by the Food and Drug Administration (FDA) for the treatment of AD [9]. Along the same lines, lecanemab represents another FDA-approved treatment for AD. Lecanemab is a monoclonal antibody that targets soluble protofibrils of Aβ and was shown to reduce Aβ levels in the brain and mitigate cognitive decline in patients with early AD exhibiting mild cognitive impairment (MCI) [105]. Importantly, this treatment has not been tested in patients with more advanced symptoms of AD. A better understanding of the physiological role of Aβ in healthy brains and the pitfalls of treatments aimed at targeting Aβ pathology is pivotal for the development of more effective and holistic treatments for AD. Importantly, the role of Aβ in the pathogenesis of AD needs to be re-evaluated, and more attention should be given to other therapeutic targets.

## 4. Tau: AD Pathogenesis, and Drawbacks of Anti-Tau Therapies

For many years, a growing number of research studies and failed clinical trials that targeted Aβ as the origin of the disease have exposed the insufficiency of the peptide in causing the disease, bringing attention to neurofibrillary tangles composed of hyperphosphorylated Tau. Besides AD, more than 20 other disorders have been identified as tauopathies, including progressive supranuclear palsy, cortico-basal degeneration, and Pick’s disease.

Tau, which stabilizes and maintains microtubule formation [106], is encoded by a single gene, *MAPT*, located on chromosome 17q21 which is translated into six human Tau isoforms derived from alternative splicing of exons 2, 3, and 10 [107,108,109,110,111]. Alternative splicing of exon 10 results in the presence of Tau isoforms containing 3- or 4-repeats (3R and 4R, respectively) of a 32-amino-acid sequence in the microtubule-binding domain (MBD), while alternative splicing of exons 2 and 4 determines the presence or absence of one or two 29-amino-acid inserts (0N, 1N, 2N) in the amino-terminal domain. Although different isoforms are expressed in different developmental stages, the adult brain expresses all six Tau isoforms with equal amounts of 3R and 4R isoforms, but unequal amounts of 1N, 0N, and 2N [112,113].

Tau can be divided into four domains as follows: (1) the N-terminal domain, (2) the proline-rich domain, (3) the MBD and (4) the C-terminal domain [114,115]. All these domains participate in microtubule-binding processes and/or microtubule assembly. The MBD contains the majority of phosphorylation sites, and phosphorylation at these sites is essential for the multifaceted function of Tau in cellular physiology. This includes the organization of the cytoskeleton via the stabilization of microtubules, maintenance of axonal stability and transport, axonal elongation and maturation, the targeting of glutamatergic receptors to postsynaptic sites, and the docking of synaptic vesicles [116]. These functions of Tau are associated with its presence in axons, the site where Tau is mainly found in adult neurons. Nevertheless, a small amount of Tau can be found in the dendrites and nuclei of neuronal and non-neuronal cells [117]. The physiological function of Tau in the dendrites is not fully understood, but it is reported to facilitate synaptic plasticity [118]. Finally, in the nucleus, Tau maintains the integrity of genomic DNA as well as cytoplasmic and nuclear RNA [117,119].

Under physiological conditions, Tau becomes phosphorylated and dephosphorylated [120]. However, under pathological conditions Tau undergoes hyperphosphorylation. Hyperphosphorylation of Tau could lead to changes in its structure, preventing its attachment to microtubules. Eventually, Tau forms insoluble aggregates named paired helical filaments (PHFs) that ultimately form neurofibrillary tangles (NFTs) [121] that can be found inside neurons and glial cells (astrocytes and oligodendrocytes). Although NFTs represent the pathological hallmarks of AD, the amount of PHFs and NFTs is only weakly correlated with the progression of the disease [122]. For instance, mice bearing the P301S Tau mutation exhibited synaptic dysfunction before the presence of NFTs [123], while suppression of the P301L mutation attenuated behavioral impairments, even though NFTs were still present [124]. The phosphorylation of Tau is important for exerting its function, and it is the result of the dynamic interplay between kinases that add a phospho-group to the protein and phosphatases that remove phospho-groups. Although several phosphatases are implicated in the dephosphorylation of Tau, the majority of studies focus on protein phosphatase 2 (PP2A), since the latter is the main responsible enzyme for Tau dephosphorylation in the human brain [125] and its activity is significantly reduced in AD brains. Post-translational modifications of the conserved catalytic subunit of PP2A and their effect on AD pathology have been widely studied [126,127]. Analysis of AD brains showed a decrease in the expression of leucine carboxyl methyltransferase (LCMT-1) that methylates PP2A and an increase in the expression of protein phosphatase methylesterase (PME-1) that demethylates PP2A [128,129,130]. In agreement with the findings above, overexpression of LCMT-1 protected against extracellularly applied Aβ-induced synaptic plasticity and behavioral deficits, while overexpression of PME-1 enhanced these impairments [131]. Importantly, the neuroprotective effect of LCMT-1 was related to decreased APP phosphorylation at the T668 site, providing a link between APP and Tau pathology [131].

Besides phosphorylation, Tau acetylation is another post-translational modification that has been extensively studied for its role in Tau’s physiological function and pathogenesis. Acetylation takes place on Lys residues and is mediated by acetyltransferases. Specifically, 27 Lys residues can be acetylated in the long isoform of Tau [132]. Acetylation of the different Lys residues could have the opposite effect on Tau aggregation. In more detail, acetylation on sites K259, K290, K321, and K353 within the KXGS motif at the MBD has been shown to be protective and inhibit Tau aggregation, while acetylation at sites K174, K274, K280, and K281 could exacerbate the pathology [133,134,135,136,137,138]. Finally, there is conflicting literature regarding the effect of increased Tau acetylation on AD pathology. Specifically, both increased and decreased Tau acetylation have been reported in AD brains. Along the same lines, animal studies have also shown confounding evidence. Transgenic mice expressing human Tau with lysine-to-glutamine mutations to mimic K274 and K281 acetylation exhibited AD-related memory deficits and impaired LTP, but in another study enhancement of p300/CBP acetyltransferase activity rescued the defects in memory and synaptic plasticity in a THY-Tau22 mouse model of tauopathy [136,139].

Considering the poor correlation between synaptic and memory decline with NFTs, the scientific community has sought to identify the toxic Tau species, determining that, as with Aβ, soluble Tau oligomers (oTau) are responsible for a series of pathological events that eventually lead to cognitive decline. In this respect, a great body of studies has shown that oTau is the toxic species in AD. A study from Fá et al. showed that extracellular application of different oTau species (recombinant human oTau, oTau extracted from AD human specimens, or naturally produced in mice overexpressing human Tau) could cause a rapid impairment in LTP and memory formation. Importantly, Tau monomers could not produce the same impairments [140,141]. In addition, pure preparations of oTau derived from human AD brains could cause immediate impairments in plasticity and propagate the disease in a prion-like manner when injected in wild-type mice [142]. The important role of oTau in the development of AD was highlighted via pathological evaluation of human AD brains. Specifically, it was shown that oTau was increased in the frontal cortex during the early stage of AD development, before the manifestation of clinical symptoms or the appearance of NFTs [143].

Although it was originally perceived that Tau pathology is triggered by Aβ, more recent studies unravel that Tau could act independently of Aβ. In the study by Fa’ et al., it was reported that external application of oTau or oAβ alone could cause impairments of synaptic plasticity and memory formation, suggesting that oTau or oAβ could act individually without requiring each other. In particular, these observations are important for oTau, showing that it could exert a detrimental effect ex vivo and in vivo, even in the absence of oAβ [140]. In support of this observation, it was shown that suppression of endogenous Tau in *Mapt*-KO mice could not protect against Aβ-induced impairments of LTP and memory formation or alter amyloid deposition. Additionally, long-term synaptic plasticity and memory were still impaired in *Mapt*-KO mice after exogenous application of oTau or oAβ [144]. The findings not only underscore the involvement of Tau in the progression of the disease but also indicate that Tau could impair synaptic plasticity and memory independently of Aβ.

Identification of oTau as the toxic species in the progression of AD not only contributed to a better understanding of the disease but also paved the way for the development of biomarkers aimed at the early diagnosis of AD. A study from Sengupta et al. conducted in the CSF collected from patients with mild AD, severe AD, and control individuals suggested that oTau could be used as an early biomarker in the CSF of AD individuals [145] in addition to already existing AD biomarkers, including Aβ42, phospho-Tau, and total Tau [146]. Another pilot study showed the presence of high-molecular-weight Tau species in the CSF of individuals with probable AD in comparison to healthy individuals. Noteworthy, oligomeric species were also present in the CSF of control individuals, but in significantly lower concentrations. The results not only strengthen the utilization of oTau in CSF as an early biomarker for AD but also bring to light a possible physiological role for oTau in the healthy brain, similar to oAβ [10].

Characterization of oTau as a main toxic entity in AD has inspired several studies aiming at developing therapeutic strategies targeting Tau pathology. In this respect, agents that mitigate Tau phosphorylation, either by inhibiting kinase activity or by promoting phosphatase activity, have been proposed as possible therapeutic strategies. Specifically, tideglusib, an inhibitor of GSK3β, was tested in a Phase II clinical trial in patients with mild-to-moderate AD. Despite its safe profile, exhibiting minimal side effects, it did not improve cognitive performance and thereafter was not further pursued [147]. Similarly, approaches aimed at reducing Tau acetylation have also failed. Specifically, salsalate, a small molecule that reduces Tau acetylation at K174, decreases Tau aggregation and rescues memory deficits in PS19 mice [137], has been under investigation in two clinical trials. The first trial investigated the safety and pharmacokinetics after 12 months of treatment in mild-to-moderate AD patients. Although the trial was completed in December 2021, the results have not been posted yet. The second trial did not show any evidence of efficacy after treatment for 6 months in 10 patients with Progressive Supranuclear Palsy [148]. Moreover, similar to approaches targeting Tau post-translational modifications, therapies against Tau aggregation have not yet worked. For instance, a phase III clinical trial with LMTX ended in May 2023, but the results are not yet disclosed. Finally, several other treatment approaches have been tested in animal models for combating the Tau pathology, but they did not enter clinical trials. These therapeutic strategies include microtubule stabilizers, agents that promote autophagy, as well as passive and active immunotherapy (for a review see [114]).

A better understanding of the pitfalls of treatments aimed at targeting tau pathology is needed for the development of effective treatments against AD. Importantly, similar to Aβ, the role of tau in the pathogenesis of AD needs to be re-evaluated, and more attention should be given to alternative therapeutic strategies.

## 5. Re-Evaluating the Role of APP in AD

The mechanisms by which Aβ and Tau affect synaptic plasticity and memory have become topics of intense scientific work. Nowadays, it is apparent that Aβ and Tau share several commonalities that could be also attributed to their common beta-sheet structure [149]. The common characteristics between Aβ and Tau are related to their susceptibility to being released in response to neuronal activity [81,83,150,151,152], a common spreading pattern, similar pervasiveness in neuronal and glial cells disrupting hippocampal synaptic plasticity, synaptic function, and memory impairment [142,153], as well as the interaction with APP, inducing in part neuronal toxicity [154,155,156,157,158].

Inevitably, several studies have pointed out an interplay between the action of Aβ and Tau in AD pathology, indicating that Tau’s role in specific aspects of the pathology is contingent upon the influence of Aβ. For instance, it was shown that Aβ dimers isolated from cortices of individuals with probable AD were able to induce Tau phosphorylation in sites that are relevant for AD and affect the integrity of the cytoskeleton. Interestingly, the absence of endogenous Tau prevented these impairments, while overexpression of human Tau exacerbated them [159]. A similar neuroprotective effect was observed when endogenous Tau was reduced in a transgenic mouse overproducing Aβ [160]. For instance, studies are showing that Aβ could promote Tau hyperphosphorylation and its subsequent dissociation from microtubules [159,161,162,163]. In this respect, it was shown in vitro that Aβ could cause acute post-translational changes to microtubules affecting their stability, thus triggering Tau hyperphosphorylation as a cellular response to restore normal microtubule function [164]. In an attempt to understand the mechanistic interplay between Aβ and Tau, it was shown that Aβ facilitates Tau seeding and aggregation by promoting its neuronal uptake [165,166]. Although these results are pivotal for understanding certain aspects of AD neuropathogenesis, they need to be interpreted cautiously. Specifically, these studies suggest that there is a crosstalk between Aβ and Tau pathology, not that Tau pathology is triggered by Aβ, as was established by the “Amyloid Cascade Hypothesis”. Researchers are challenging the conventional model that suggests Aβ and Tau act sequentially in the progression of AD. The studies of Fá’ et al. and Puzzo et al. provide strong evidence regarding the independent action of oTau and oAβ in inducing synaptic plasticity impairments [140,144]. Intriguingly, oTau could act in combination with oAβ to impair memory and LTP, as the combination of sub-toxic doses of oTau and oAβ impairs both LTP and memory [140]. Although these results do not rule out the possibility that Aβ mediates certain aspects of its excitotoxic functions through Tau, they provide evidence of the parallel and independent action of Aβ and Tau, opposing the previous notion suggesting that Aβ acts through Tau to impair plasticity and memory in AD. In this respect, Aβ- or Tau-centric therapies cannot be fully successful because they target only one aspect of the pathology, while the other could still be present propagating the disease. Thereafter, therapies that act on both Aβ and Tau represent a more promising therapeutic option. Nevertheless, a combination of therapies might not be possible considering the important physiological role of these two proteins in the healthy brain [89,114]. Hence, therapies that act on a downstream common effector might represent the most successful and physiologically relevant approach. Indeed, the observation that a combination of subtoxic doses of oTau or oAβ leads to LTP and memory decline suggests that these two proteins act on one or multiple common targets [140].

Although several intracellular cascades seem to represent a converging point between Aβ and Tau, more recent studies have been dedicated to the important role of APP in mediating Aβ and Tau synaptotoxicity. Importantly, both Aβ and Tau interact and bind directly to APP. It was shown that APP homodimers at the presynaptic cell could act as receptors for Aβ binding, promoting glutamate-induced excitotoxicity [157]. Regarding the interaction between APP and Tau, it was shown several years ago that there is a Tau-binding domain associated with FAD within the APP molecule [167,168,169]. These observations were strengthened by a more recent study by Puzzo et al. showing that both oTau and oAβ could bind directly to APP [170]. Accordingly, the presence of APP was required for the oAβ- and oTau-induced impairments of synaptic plasticity and memory, since APP-KO were resistant to these types of impairments after extracellular application of either oAβ or oTau.

These findings raise the question of how APP mediates Aβ or Tau excitotoxicity. Although it seems that there is a lack of understanding regarding the complicated relationship between APP and the toxic forms of Aβ and Tau, some pieces of the puzzles have already been revealed. An important finding is that APP presence is a requirement for oAβ and oTau internalization within the neuronal cells. Interestingly, when it comes to oTau, it was shown that its internalization by astrocytes is APP-dependent; upon entering, oTau impairs the metabolism of astrocytes inducing deficits in neuronal plasticity [171]. Nevertheless, it is still not clear whether APP is needed for the internalization of oAβ or oTau into the cells per se, or whether binding of oAβ or oTau to APP could affect second messenger cascades that will subsequently have a deleterious impact on synaptic plasticity and memory. Although these concepts are not mutually exclusive, previous work has demonstrated that at least for certain aspects of neuronal plasticity impairments, intraneuronal accumulation of Aβ is required [172]. Moreover, intraneuronal accumulation of toxic Aβ has been associated with the induction of cellular stress in neurons before the appearance of cognitive deficits [173]. Similarly, the accumulation of intraneuronal Tau is associated with impairments in mitochondrial function and autophagy deficits that subsequently impair normal neuronal function [173,174].

The mechanism by which APP might promote the entry of oAβ or oTau into the cell is still unclear, but a few mechanisms have been hypothesized. There is the possibility that oAβ and oTau enter the neurons via endocytosis along with APP. In this respect, it is known that both oAβ and oTau physically interact with APP and that APP enters neurons via endocytosis [175]. Another possible scenario is that APP itself forms dimers that act as channels to allow the entry of small molecules to enter the cell. The expression of APP in *Xenopus* oocytes and the external application of its fragment containing the Aβ sequence induced membrane ion currents reminiscent of ion channel activation [176]. Alternatively, oAβ and oTau could enter the neurons via pores in the membranes. For instance, it was reported that soluble Aβ species could increase the conductivity of the plasma membrane, altering its permeability [177]. Although the implication of APP in this process is not evident, it is possible that the binding of oAβ or oTau to APP could alter the integrity of the membrane to facilitate the formation of pores.

The binding of oAβ or oTau to APP could lead to conformational changes that would eventually manifest in synaptic plasticity and memory deficits. In this respect, it was shown that the binding of oAβ or oTau to APP does not cause toxicity by triggering the amyloidogenic pathway, since blockage of β-secretase activity in BACE-KO mice did not protect against LTP impairments after extracellular application of either oAβ or oTau [170]. Thereafter, it is possible that the binding of Aβ and Tau oligomers could cause structural changes in the molecule, preventing interaction with other proteins, and shifting its signaling from neuroprotective to neurotoxic. This could happen either by altering the signaling pathways mediated by AICD/AID or by post-translational changes in the APP. It was recently shown that APP-mediated expression of the heparan sulfate proteoglycan (HSPG), glypican 4 (GPC4), facilitates entry of oTau in astrocytes [178]. Specifically, AICD/AID enters the nucleus, where it binds to the promoter of *gpc4*, enhancing expression of the GPC4 receptor at the membrane. Subsequently, GPC4 facilitates the internalization of extracellular oTau to the astrocytes [178], corroborating previous findings showing that GPC4 facilitates Aβ uptake from neuronal stem cells [179]. Of note, a reduction in GPC4 expression was observed in the brains of APP-KO mice or APP-TA mice in which the T668 site cannot be phosphorylated, underscoring the importance of T688 phosphorylation in AICD/AID production and eventually oTau internalization [178].

**Figure 1:**
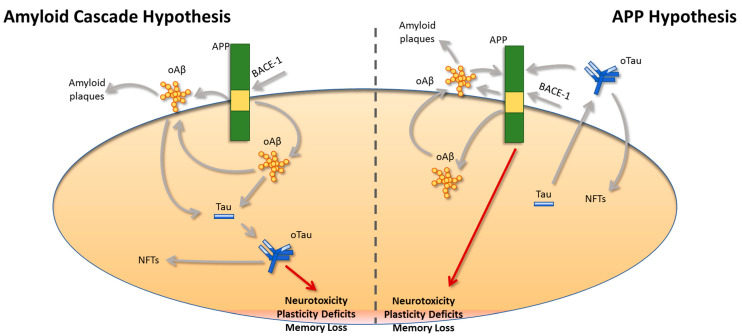
A revised view of the relationship between APP, Aβ, and Tau. Based on the “Amyloid Cascade Hypothesis”, amyloid-beta (Aβ) peptides generated after cleavage of the amyloid precursor protein (APP) by β-secretase-1 (BACE-1) are the main toxic species in Alzheimer’s disease (AD). Based on this original view regarding the pathogenesis of AD, Aβ peptides will trigger Tau pathology, leading to the progression of the disease due to neuronal loss, plasticity, and memory deficits. This view was updated after the discovery that oligomeric Aβ (oAβ) and oligomeric Tau (oTau) are responsible for the neurotoxicity in AD, rather than their insoluble deposits referred to as amyloid plaques and neurofibrillary tangles (NFTs), respectively. Nevertheless, more recent findings challenge this hypothesis and suggest that the pathogenesis of oAβ and oTau occurs independently and not in sequence as was suggested by the “Amyloid Cascade Hypothesis”. Specifically, our revised hypothesis proposes that oAβ and oTau act in parallel and via APP to induce plasticity and memory deficits. Both oAβ and oTau bind to APP, and their expression is important for the intraneuronal uptake of both peptides, rendering APP a common target for both oAβ and oTau.

Collectively the data presented above highlight that Aβ and Tau act independently of each other in parallel and not in sequence, as was previously proposed by the ‘Amyloid Cascade Hypothesis’ (Figure 1). Additionally, the identification of APP as a common target for both Aβ and Tau brings new insights into our understanding of the pathogenesis of AD, possibly opening new avenues for therapeutic interventions. Considering the pitfalls of the existing treatment approaches, finding new targets for combating AD is of great importance. In this respect, identification of the exact function of the APP molecule and the precise mechanism that mediates Aβ and Tau neurotoxicity should shed light on the complicated interplay between APP, Aβ and Tau. Notably, unraveling whether APP acts as a receptor or a channel at the plasma membrane has been a matter of intense discussion [180]. From a drug discovery perspective, targeting APP per se could lead to disturbances of normal physiological processes, since APP mediates several functions in the central nervous system. Nevertheless, other options could be explored aiming to mitigate pathological changes that will render the function of APP synaptotoxic. For instance, phosphorylation at the T688 site has been studied for its implication in AD, and it was shown that blocking phosphorylation at this site could alleviate synaptic plasticity and memory impairments induced by oAβ as well as reducing oTau entry in astrocytes.

In this respect, a few approaches have been proposed for reducing T688 phosphorylation and alleviating cognitive impairments in AD [181]. For instance, insulin and insulin-like growth factor-I (IGF-I) reduced T688 phosphorylation in neuronal cultures from healthy rats and in a mouse model of insulin resistance and metabolic syndrome that exhibited cognitive decline [182]. This effect was mediated by the phosphatidylinositol 3- kinase (PI3-K)/protein kinase B (Akt) pathway [182]. Nevertheless, insulin resistance abolished the effect of IGF-I on APP phosphorylation at T688, suggesting that treatment with insulin and IGF-I cannot be beneficial in AD patients with insulin resistance, metabolic syndrome, or diabetes [182]. Interestingly, intranasal administration of insulin seemed to have a beneficial effect on cognitive function in a subset of patients with AD and MCI, encouraging the development of more comprehensive clinical studies [183]. Another line of research has shown that the death-associated protein kinase 1 (DAPK1) could trigger phosphorylation of T688. Genetic variations in *DAPK1* have been associated with late-onset AD [184] and DAPK1 expression was increased in the hippocampus of AD patients, as compared to age-matched healthy individuals [185]. Additionally, DAPK1 overexpression was linked with aberrant Tau phosphorylation [185] and increased secretion of Aβ from neuronal cell cultures [186]. DAPK1 was also shown to increase the phosphorylation of APP at T688, promoting amyloidogenic cleavage of APP. In an AD mouse model, KO of DAPK1 reduced T688 phosphorylation and Aβ generation, suggesting that targeting DAPK1 could have a therapeutic potential for AD [186].

Another interesting target that has been explored for its therapeutic relevance in AD is the dual-specificity tyrosine phosphorylation-regulated kinase 1A (DYRK1A). Recent studies suggest that overexpression of DYRK1A could lead to plasticity and memory impairments in mice [187,188], while inhibition of the peptide is emerging as a possible treatment for several neurodegenerative disorders [189]. With regards to AD, DYRK1A was shown to enhance the phosphorylation of APP at T688, promoting its amyloidogenic cleavage and the production of Aβ40 and Aβ42 [190,191]. In turn, Aβ was shown to facilitate DYRK1A activity, extenuating its negative impact on APP cleavage [192]. Additionally, DYRK1A can phosphorylate Tau in several AD-relevant epitopes [193,194]. In this respect, chronic DYRK1A inhibition ameliorated cognitive impairments in an AD mouse model and furthermore resulted in decreased APP phosphorylation, reduction of Aβ production, and Tau phosphorylation in the insoluble fragment, while NFT pathology remained unaltered [195,196]. These results underscore the importance of targeting molecules that reduce T688 phosphorylation, due to their beneficial effect in reducing Aβ and Tau pathology. Undoubtfully, our suggested model that oAβ and oTau act through APP to propagate the disease and exert their deleterious effects in synaptic plasticity and memory could pave the way for a new era of studies aiming at unraveling the complex role of APP in AD pathology, with the ultimate goal of developing efficacious therapeutic approaches for the treatment of AD.
